# Microfluidic Lab-on-a-Chip Based on UHF-Dielectrophoresis for Stemness Phenotype Characterization and Discrimination among Glioblastoma Cells

**DOI:** 10.3390/bios11100388

**Published:** 2021-10-13

**Authors:** Elisa Lambert, Rémi Manczak, Elodie Barthout, Sofiane Saada, Elena Porcù, Francesca Maule, Barbara Bessette, Giampietro Viola, Luca Persano, Claire Dalmay, Fabrice Lalloué, Arnaud Pothier

**Affiliations:** 1 XLIM-UMR 7252, University of Limoges/CNRS, 87060 Limoges, France; elisa.lambert@xlim.fr (E.L.); remi.manczak@xlim.fr (R.M.); claire.dalmay@xlim.fr (C.D.); 2 CAPTuR-EA 3842, University of Limoges, 87025 Limoges, France; elodie.barthout@unilim.fr (E.B.); sofiane.saada@unilim.fr (S.S.); barbara.bessette@unilim.fr (B.B.); fabrice.lalloue@unilim.fr (F.L.); 3 Department of Women’s and Children’s Health (DSB), University of Padova, 35128 Padova, Italy; elena.porcu@unipd.it (E.P.); giampietro.viola.1@unipd.it (G.V.); luca.persano@unipd.it (L.P.); 4 Institute of Pediatric Research (IRP), 35127 Padova, Italy; 5 Arnie Charbonneau Cancer Institute, Department of Biochemistry and Molecular Biology, University of Calgary, Calgary, AB T2N 1N4, Canada; francesca.maule@ucalgary.ca

**Keywords:** high-frequency dielectrophoresis, glioblastoma cells, single cell manipulation, microfluidic point-of-care device, cancer stem cells

## Abstract

Glioblastoma (GBM) is one of the most aggressive solid tumors, particularly due to the presence of cancer stem cells (CSCs). Nowadays, the characterization of this cell type with an efficient, fast and low-cost method remains an issue. Hence, we have developed a microfluidic lab-on-a-chip based on dielectrophoresis (DEP) single cell electro-manipulation to measure the two crossover frequencies: *f_x01_* in the low-frequency range (below 500 kHz) and *f_x02_* in the ultra-high-frequency range (UHF, above 50 MHz). First, in vitro conditions were investigated. An U87-MG cell line was cultured in different conditions in order to induce an undifferentiated phenotype. Then, ex vivo GBM cells from patients’ primary cell culture were passed through the developed microfluidic system and characterized in order to reflect clinical conditions. This article demonstrates that the usual exploitation of low-frequency range DEP does not allow the discrimination of the undifferentiated GBM cells from the differentiated one. However, the presented study highlights the use of UHF-DEP as a relevant discriminant parameter. The proposed microfluidic lab-on-a-chip is able to follow the kinetics of U87-MG phenotype transformation in a CSC enrichment medium and the cancer stem cells phenotype acquirement.

## 1. Introduction

Glioblastoma (GBM) is the most frequent and highly malignant brain tumor in adulthood classified as a high-grade glioma (grade IV), considered as the most aggressive tumors of the central nervous system. Worldwide, 240,000 brain tumors are diagnosed each year, the majority of which are GBM [1]. GBM is associated with a poor prognosis with a mean survival of 12 months. Indeed, standard treatment such as surgery and combined radio–chemotherapy [2] fails to improve patients’ care [3]. Despite recent advances in targeting therapies and immunotherapies, the current treatments do not allow the improvement of the mean survival. This very poor prognosis is mainly due to frequent relapses, despite the regression or disappearance of the tumor upon the golden standard treatment, i.e., the Stupp protocol [2]. Consequently, this pathology is very difficult to handle.

The high recurrence of GBM can be explained by the high heterogeneity of cellular, genetic and morphological patterns of the cell populations present in the tumor [4], which alters the efficiency of conventional therapies. This cell heterogeneity mainly results from a small cell subpopulation, called cancer stem cells (CSCs) [4], which are now considered as one of the major factors responsible for tumor progression and relapse [5]. In fact, CSCs display an immature and undifferentiated phenotype associated with specific self-renewal features. They also are likely to regenerate the entire tumor. Moreover, due to their quiescent properties, CSCs are resistant to radio and chemotherapy targeting proliferating cells [1]. Thus, CSCs detection in solid tumor could afford a prognosis value to evaluate tumor aggressiveness and prevent recurrence risk. However, CSCs are a rare cell subpopulation difficult to characterize with specific and usual tumor biomarkers within the tumor. CSC detection currently represents a challenge to improve the management of certain solid cancers, in particular GBM. Thus, new approaches for effectively discriminating CSCs from differentiated tumor cells are regularly investigated. However, one of the main encountered difficulties in characterizing CSCs is the lack of specific markers for these cells.

In this objective, biologists commonly analyze a panel of biomarkers by using conventional approaches for characterizing CSCs such as immunofluorescence, flow cytometry and protein array analysis. These different methodologies require systematic immunolabeling to identify CSCs, thereby require multiple steps which increases both expenses and inconvenience. Indeed, these methodologies are time-consuming and additional costs are incurred for the purchase of specific antibodies against CSC biomarkers. In addition, immunolabeling can influence and modify cell behavior and differentiation mechanisms. These changes might affect cell cultures and limit further analyses [6]. In order to overcome these issues and avoid immunolabeling, researchers started to develop alternative label-free methods for cell characterization. These innovative technologies aim to characterize CSCs based on their specific physical properties. Thus, most of the recent techniques rely on an external force coupled with microfluidics. This allows the reduction of the cost and analysis time with the possibility of parallelization and the reduction of sample volume as only few µL are needed. It also prevents cells from damage by limiting mechanical stress. Among the label-free method, dielectrophoresis (DEP) presents an important interest as it allows the investigation of biological cell behavior according to its intrinsic dielectric properties. For this study, we use DEP electro-manipulation at low frequency (below 500 kHz) and at ultra-high-frequency (UHF) range (above 50 MHz), highlighting the relevance of using UHF-DEP phenomenon to discriminate undifferentiated cells (CSC) from differentiated tumor cells. Implementation of the proposed microfluidic lab-on-a-chip is performed on BiCMOS technology and allows the screening of the intracellular properties of GBM cells.

### 1.1. Basic DEP Theory

Dielectrophoresis is a physical phenomenon, which leads to the motion of a polarizable particle such as biological cells, in a non-uniform electric field due to the interaction between the induced dipole of the particle and the field gradient. The polarization phenomenon redistributes of the charges at the interface between the particle and the suspension medium. The dielectrophoretic force exerted on the polarized particle in a non-uniform electric field is expressed as follows [7]:

(1) FDEP=2πr3εmRe[fCM(ω)] ∇E2 

where r is the radius of the particle, $\varepsilon_{m}$ the permittivity of the suspension medium, Re[*f_CM_*(ω)] the real part of the Clausius–Mossotti (CM) factor, E the applied electric field.

**Figure 1:**
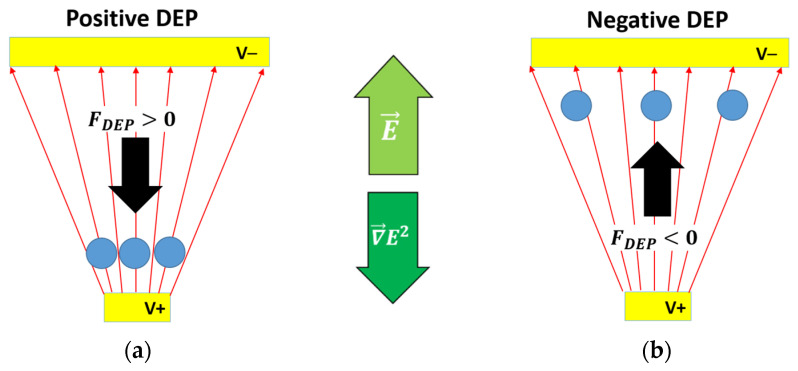
Particles’ interaction in a non-uniform applied field: (**a**) the DEP force is positive, i.e., collinear to the electric field gradient ∇E², and the particles are attracted to areas of high field intensity (positive DEP); (**b**) the DEP force is negative, i.e., opposite to the electric field gradient ∇E², and the particles are repelled toward areas of low field intensity (negative DEP).

The Clausius–Mossotti factor describes the polarization state of a particle in a suspension medium. It depends on the dielectric properties (permittivity and conductivity) of the medium and the particle [7]:

(2) $$f_{CM}(\omega )=\frac{\varepsilon_{p}^{*}-\varepsilon_{m}^{*}}{\varepsilon_{p}^{*}+2\varepsilon_{m}^{*}}$$

where $\varepsilon_{p}^{*}$ and $\varepsilon_{m}^{*}$ are the complex permittivity of the particle and the medium, respectively. The complex permittivity can be defined as:

(3) $$\varepsilon^{*}=\varepsilon \;-\;j\;\frac{\sigma }{\omega }\;$$

where ε is the absolute permittivity (ε = ε_r_*ε_0_, with ε_r_ is the relative permittivity and ε_0_ is the vacuum permittivity, of which the value is 8.854 F·m^−1^), σ the conductivity and ω the angular frequency of the electric field. The sign of the real part of the CM factor determines the orientation of the DEP force. In Figure 1a, when Re[*f_CM_*(ω)] is positive, the DEP force attracts the particle to the strong field areas. This phenomenon is called positive DEP (pDEP). In Figure 1b, when Re[*f_CM_*(ω)] is negative, the DEP force is then repulsive and the particle is repelled towards the weak electric field areas. This is called negative DEP (nDEP).

From a physic point of view, a biological cell can be modelized as a spherical dielectric particle that is submitted to the DEP force. A cell is a complex biological object, but it can be properly modeled into a simpler single-shell model with a reduced number of dielectric parameters associated to each component of the cell.

### 1.2. From a Biological Cell to a Single-Shell Model

**Figure 2:**
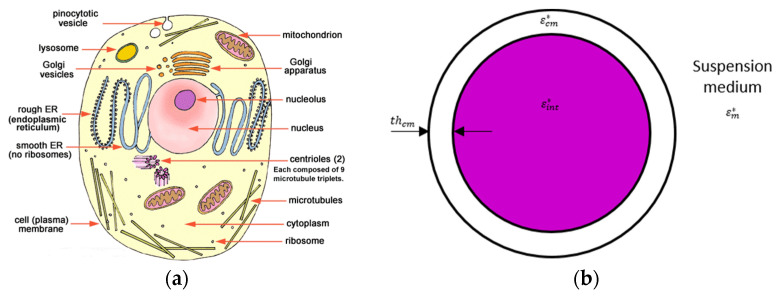
(**a**) Representation of a biological cell in a suspension medium; (**b**) its single-shell model with $\varepsilon_{\mathrm{int}}^{*}$ the complex permittivity of the cellular content, $\varepsilon_{\mathrm{cm}}^{*}$ the complex permittivity of the cell membrane and ${\mathrm{th}}_{cm}$ the thickness of the cell membrane and $\varepsilon_{m}^{*}$ the complex permittivity of the suspension medium.

In order to predict cells’ behavior with an applied electric field, it is helpful to have a simplified model of a biological cell. The Figure 2 presents a schematic of a cell and its commonly used associated single-shell model associated where the cell membrane and cytoplasm are represented by a shell and a core with their own complex permittivity [8,9,10].

Indeed, the single-shell model considers the cytoplasm and its content as a homogeneous dielectric sphere enveloped by the plasma membrane. This model limits the number of dielectric parameters to take into account only the complex permittivity of the intracellular content, the cell membrane and the suspension medium. Actually, the Clausius–Mossotti factor depends on these parameters as well as the frequency of the applied field. This single-shell model will be considered in this paper.

### 1.3. Effect of the Cellular Dielectric Properties on the Clausius–Mossotti Factor

**Figure 3:**
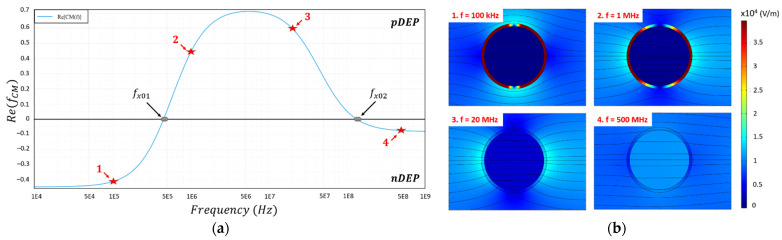
Numerical simulation of a cell dielectric behavior in function of the frequency with the parameters from Table 1. The design used for the biological cell is the single-shell model. (**a**) Numerical simulation of the real part of the Clausius–Mossotti factor. The red stars correspond to the chosen frequencies for the COMSOL simulation; (**b**) COMSOL simulation of the single-shell model for different frequencies (100 kHz; 1 MHz; 20 MHz; 500 MHz) which correspond to the curve of the CM factor. The color scale corresponds to the electric field intensity (V/m) and the black lines correspond to the electric streamlines.

Figure 3 illustrates the frequency-dependent cell behavior through the real part of the CM factor (Figure 3a). The dielectric parameters and cell geometric parameters are reported in Table 1. The real part plot in Figure 3a is computed thanks to the myDEP software [11]. nDEP behavior can be observed at very low frequency (lower than 400 kHz) and at high frequency (at least higher than 150 MHz), and pDEP behavior can be seen at medium range frequency (between 500 kHz and 100 MHz). The plot of the CM factor hence presents alternations between a repulsive state (nDEP) and an attractive state (pDEP). Two crossover frequencies *f_x01_* and *f_x02_* appear where the real part of the CM factor becomes null. *f_x01_* occurs at low frequency, whereas *f_x02_* occurs at higher frequency.

Moreover, from Figure 3a, 100 kHz and 1, 20 and 500 MHz frequencies were selected in order to study the dielectric response of the cells. Indeed, these frequencies correspond to the two different DEP behaviors but at low frequency (frequencies n°1 and n°2) and at high-frequency regime (frequencies n°3 and n°4). COMSOL Multiphysics^®^ computations were performed with the AC/DC electric current module in Figure 3b. The parameters from Table 1 were used for the simulation in such a way that the results correspond to the curve of the real part of the CM factor. As said before, the cell is represented by the single-shell model with the core: its intracellular content, and the shell: its plasma membrane. The cell is here considered to be suspended in a low-conductivity medium. The electric potential is 1 Vpp. The shown colors represent the electric field intensity in V/m from dark blue (the field intensity is 0 V/m) to dark red (the field intensity is maximum). One can notice that for the nDEP behavior (at 100 kHz and 500 MHz), the electric field lines (black streamlines) bypass the cell, whereas for the pDEP behavior (at 1 and 20 MHz), the electric field lines seem attracted inside the cell. This is mainly due to the reorientation of the charges at the interface between the cell membrane and the medium [7]. One can also notice that at low frequency, no field can reach the cell content. The electric field is at maximum inside the plasma membrane, which hence acts as an insulator. As a result, at low frequency, the electromagnetic field will be more sensitive to the physical and dielectric properties of the cell membrane. The more the frequency of the applied signal increases, the more the electric field can penetrate inside the cell and starts to interact with and so probe the cellular content. Consequently, at high frequency, the electromagnetic wave can be deeply sensitive to the intracellular content.

Moreover, it is possible to change the value of the dielectric parameters in order to study the evolution of the real part of the CM factor and the parameter dependency of the two crossover frequencies as has been carried out in [12,13,14]. A first approximation of the crossover frequency *f_x01_* can be expressed as [7]:

(4) $$f_{x01}{=\sigma }_{m}\;\frac{{\mathrm{th}}_{\mathrm{cm}}\;}{\sqrt{2}{\pi \;r\;\varepsilon }_{\mathrm{cm}}\;}$$

The crossover frequency *f_x01_* depends mostly on the dielectric parameters of the plasma membrane, but also the particle radius. Hence, *f_x01_* is more sensitive to the cell shape, its morphology and to the plasma membrane properties. It has been widely used to separate cells or polystyrene particles of different size [15,16] and to separate living from non-viable cells [17]. The second crossover frequency *f_x02_* can be approximated with the assumption that the conductivity of the suspending medium (20 mS/m, see Section 2) is significantly below the intracellular value expression [18]:

(5) $$f_{x02}=\frac{\sigma_{\mathrm{int}}}{2\pi \;}\;\sqrt{\frac{1}{2\varepsilon_{m}^{2}\;{-\;\varepsilon }_{\mathrm{int}}\varepsilon_{m}\;-\;\varepsilon_{\mathrm{int}}^{2}}}$$

The crossover frequency *f_x02_* depends on the dielectric parameters of the intracellular content [19]. Hence, cells can be individually electro-manipulated by the DEP force motion according to their own dielectric properties of their cytoplasm. As an example, UHF-DEP has already been successfully used in order to discriminate differentiated from undifferentiated medulloblastoma cells [20,21].

This paper aims to show the relevance of the identification of the crossover frequency *f_x02_* as the DEP signature and then using it as an appropriate discriminant biomarker to detect CSCs within tumor cell population. Therefore, the two crossover frequencies *f_x01_* and *f_x02_* have been measured for each investigated GBM cell and compared.

## 2. Materials and Methods

### 2.1. Cell Line Culture

Human GBM cell line U87-MG was purchased from American Type Culture Collection (ATCC). Cells were grown in different culture conditions (see below) at 37 °C in a humidified atmosphere of 5% CO_2_–95% air. Cancer stem cells enrichment was obtained submitting cells to stringent culture conditions with the Define Medium (DM). Two culture conditions were used for the cells’ DEP characterization:

- Normal Normoxia Medium (NM): induces normal differentiation in DMEM supplemented by 10% FBS, 2 mM glutamine and 1% penicillin/streptomycin.
- Define Normoxia Medium (DM): the starvation of 10% Fetal Bovine Serum (FBS) in this medium induces stringent conditions. DM is supplemented in two specific growth factors: EGF (Epidermal Growth Factors) and bFGF2 (basic Fibroblast Growth Factors) required for clonal expansion and the formation of glioma spheres which are composed of several thousand aggregated cells. DM composition consists in DMEM/F12 supplemented by 0.6% glucose, 1% sodium bicarbonate, 1% MEM non-essential amino acids, 5 mM HEPES, 9.6 µg/mL putrescine, 10 µg/mL ITSS, 0.063 µg/mL progesterone, 60 µg/mL *N*-acetyl-l-cysteine, 2 µg/mL heparin, 0.1 mg/mL penicillin/streptomycin, 50X B-27 supplement without vitamin A, 20 ng/mL EGF, 20 ng/mL bFG.

Hence, it is expected that the Define Medium will select undifferentiated cells and also promote the emergence of CSCs, whereas the Normal Medium will induce a differentiation of cells and so will result in a very low ratio of cancer stem cells. For the differentiated cells, they were cultured for 6 days in NM before the DEP characterization. For the undifferentiated cells, they were cultured for 5 days in DM or maintained during 21 days in DM in order to study the kinetics of CSC appearance and the emergence of stem cell characteristics.

### 2.2. Primary GBM Cell Isolations, Culturing and Separation by Flow Cytometry

For some validation experiments, four different primary cell cultures have been isolated and established in vitro from GBM patients to characterize any potential difference in term of DEP signature between bulk cells and their relative CSCs counterparts. Written informed consent for the donation of adult tumor brain tissues was obtained from patients before tissue collection under the auspices of the protocol for the acquisition of human brain tissues obtained from the Ethical Committee of the Padova University Hospital (2462P). In particular, GBM cells were isolated from tumors during surgery as previously described [22]. Briefly, resected GBM samples were dissociated, and single cell suspensions were grown in Define Medium under hypoxic conditions (DH). Indeed, GBM cells were maintained in an atmosphere of 2% oxygen, 5% carbon dioxide and balanced nitrogen in a H35 hypoxic cabinet (Don Whitley Scientific Ltd., Shipley, UK) to achieve a proper expansion of the CSC subpopulation in hypoxic conditions mimicking the GBM microenvironment [23].

After proper expansion in vitro, primary GBM cells were incubated with a PE mouse anti-CD133 (AC133) antibody (Miltenyi Biotec, Bergisch Gladbach, Germany) according to manufacturer’s indications and then separated into a CD133+ and a CD133− subpopulation by means of Fluorescence Activated Cell Sorting (FACS) in a MoFlo XDP cell sorter (Beckman Coulter, Brea, CA, USA). A CD133 versus side scatter dot plot revealed the populations of interest characterized by the expression (or not) of the CD133 marker. Cell fractions were selected by setting appropriate sorting gates as previously described [24]. Upon sorting, isolated GBM cell subpopulations (CD133+ and CD133−) were washed in Phosphate Buffered Saline (PBS), frozen in medium containing 10% DMSO and then cryopreserved in liquid nitrogen for subsequent thawing and DEP characterization.

### 2.3. DEP Suspension Medium

Few minutes from the DEP characterization, U87-MG cells were suspended in an osmotic medium adapted for DEP electro-manipulation made from deionized water (ion free) supplemented by sucrose. The conductivity of the DEP medium is 26 mS/m and the pH is 7.4. The crossover frequency measurements were performed at room temperature.

### 2.4. Comparative Transcriptomic Analysis (mRNA Levels) of the Stemness Phenotype

In order to confirm the enrichment of undifferentiated cells in Define Medium (DM), a comparative transcriptomic analysis of the stemness phenotype was performed.

The extraction of the total RNA from U87-MG cell line was carried out using the RNeasy kit (Qiagen) on 1 million cells according to the manufacturer’s recommendations. Quantitative Polymerase Chain Reaction (qPCR) was performed on 50 ng of cDNA using Taqman probes on *GAPDH* and *HPRT* as reference genes. The CSC markers used are *CD133*, *Nanog*, *Sox2* and *Oct4*. The analysis is performed using QuantStudio3 (Thermo Fisher) and relative expressions are estimated by ΔΔCt method using the average of the two reference genes as endogenous control.

### 2.5. Crossover Frequency Experiment

#### 2.5.1. DEP Sensor Design and Experimental Setup

**Figure 4:**
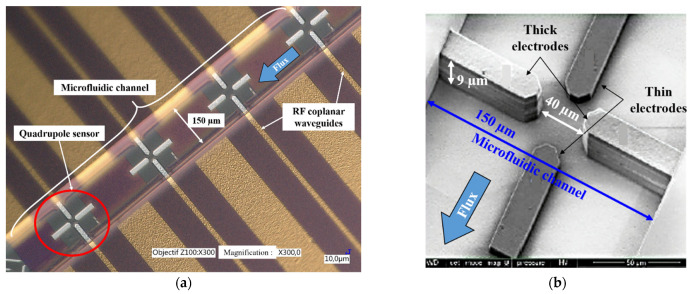
(**a**) Quadrupole microelectrodes sensor implemented with a microfluidic channel in BiCMOS; (**b**) Scanning Electron Microscopy (SEM) picture of a quadrupole sensor.

In order to identify the cells’ DEP signature, we use a specific BiCMOS RF-sensor implemented on a microfluidic chip, as presented in Figure 4. The developed UHF-DEP lab-on-chip allows the electro-manipulation of one single cell. Its structure is made of four electrodes to generate a non-uniform electric field. They are set at 90° across the microfluidic channel. In order not to disturb the fluid flow and not to obstruct the channel with the passage of cells, the two electrodes parallel to the channel (in dark gray in Figure 4b) are very thin and 0.45 µm high. The other pair of electrodes perpendicular to the channel are thicker: 9 µm high, in order to ensure a sufficiently strong field over the height of the channel. The implemented gaps between the electrodes are 40 µm wide to generate dielectrophoretic force with a low applied voltage to trap efficiently biological cells. The two pairs of electrodes are biased with a high-frequency continuous wave (CW) signal. The fabrication process of the chip is detailed in [25]. The microfluidic channel is molded in a polydimethylsiloxane (PDMS) cap to drive the cell suspension to the sensor array. The channel is 150 µm wide and 50 µm high.

**Figure 5:**
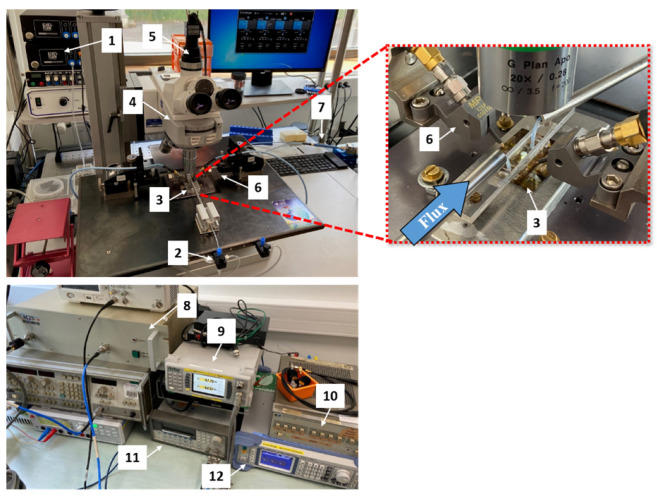
Experimental bench where the crossover frequencies measurements are performed. The labeled parts refer to: (**1**) external flow controllers Elveflow OB1; (**2**) cell suspension; (**3**) UHF-DEP microfluidic chip, a zoomed picture is shown in the red dotted box; (**4**) Scope.A1 Zeiss Microscope; (**5**) camera Axiocam 105 color Zeiss; (**6**) RF probes MPI TITAN T26P-GSG-150; (**7**) power divider; (**8**) power amplifier Bonn Elecktrik BLWA 100-5M; (**9**) power meter Anritsu ML2496A; (**10**) attenuator/switch driver 11713A HP; (**11**) low-frequency generator Agilent 33250A; (**12**) RF signal generator SMB 100A from Rhode & Schwarz.

The experimental setup for the crossover frequency measurement is shown in Figure 5. Once the cells were suspended in the DEP medium, the Eppendorf was linked to the UHF-DEP lab-on-chip thanks to capillary tubes. The cell suspension is injected in the chip by external flow controllers. They apply input and output pressures in order to regulate the speed and the motion of the cells in the microfluidic channel.

The UHF signal is produced thanks to a radio-frequency signal generator (whose frequency range is adjustable from 10 MHz to 1.1 GHz) which is then amplified. The signal generated can reach a magnitude of 10 Vpp while keeping a high purity continuous wave (CW) signal. During the crossover frequency measurement, the signal voltage is set between 2 and 4 Vpp. The applied signal is then directed to a power divider in order to bias the pair of thick electrodes simultaneously with the same signal, while the thin electrodes are grounded. The DEP signal is propagated until the quadrupole sensor thanks to 50 Ω microstrip transmission lines which are connected to RF probes. The switch driver allows switching between the high-frequency applied signal to the low-frequency applied signal. To measure the first crossover frequency *f_x01_*, a low-frequency signal can be generated by a second generator whose frequency range can be set from 1 μHz to 80 MHz. Then, the low frequency applied signal is set between 2 and 4 Vpp and propagated through the power divider to the RF probes.

#### 2.5.2. *f_x01_* and *f_x02_* Crossover Frequencies Measurements

**Figure 6:**
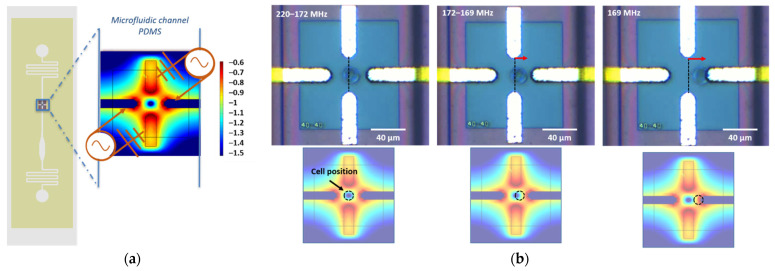
Principle of the crossover frequency measurement: (**a**) schematic of the microfluidic chip with a zoom in on one quadrupole sensor. Computation of the biased sensor in a non-uniform electric field (COMSOL Multiphysics^®^). The scale color corresponds to the normalized electromagnetic field intensity; (**b**) dielectrophoretic response of a single U87-MG NM cell under an UHF applied signal for frequencies between 220 and 169 MHz. The second crossover frequency *f_x02_* is measured at 169 MHz.

The aim of this article is to show that we can take benefit from the second crossover frequency *f_x02_* to discriminate differentiated cells from undifferentiated cells in GBM cell line and patient GBM cells from primary culture, whereas *f_x01_* cannot emphasize this discrimination. To do so, we used our lab-on-chip to measure the crossover frequencies and to characterize cells’ DEP signature according to their different culture conditions (Normal Medium NM and Define Medium DM). First, the cells are brought to the characterization area, i.e., the quadrupole sensor, thanks to the external flow controllers. Once a single cell is present in the center of the quadrupole such as in the first picture of Figure 6b, the flow is temporarily cut off and stabilized in order to proceed to the crossover frequency measurement and the electromagnetic signal is switched on. The flow is stopped during the DEP characterization to avoid the competition between forces, so that the cell is only submitted to the dielectrophoretic force and the natural gravity.

The Figure 6a shows the quadrupole biased whatever the investigated frequency from the low- to high-frequency range. One can notice that the areas of strong electric field intensity (in orange/red) are located at the different edges of the electrodes. As said before, these zones are related to the pDEP cell behavior, whereas the area of weak field intensity (in dark blue) is located at the center of the electrodes, which is assimilated to the nDEP cell behavior. The DEP sensor is biased firstly with the UHF generated signal at 500 MHz. At this frequency range (Figure 3a), we expect the cell to present nDEP behavior, and it is far from its crossover frequency. The dielectrophoretic force is thus repulsive and the cell is trapped within the central electrical cage created by the quadrupole, as shown in the first picture of Figure 6b. Then, we decrease the frequency of the applied signal. The DEP force starts to become attractive and we can observe the first movement of the cell (second picture in Figure 6b). Finally, the cell is pulled toward the edge of one of the lateral electrodes, which is the pDEP area (last picture in Figure 6b). Hence, we can tune the frequency of the signal from a repulsive state in the center of the sensor to an attractive state. The crossover frequency *f_x02_* can be determined from the motion of the cell from the nDEP behavior to the pDEP behavior, which can be observed optically under a microscope. In order to precisely identify *f_x02_*, we first decrease the frequency of the applied signal by steps of 10 MHz in order to approach the crossover frequency. Then, we slowly scan the frequency by steps of 1 MHz to observe the cell motion. This operation is repeated once again in order to accurately determine *f_x02_*. Then, we increase the applied frequency to place the cell in the center of the quadrupole. We turn off the UHF signal generator and use the switch driver in order to inject the low-frequency signal in the lab-on-a-chip and to determine the first crossover frequency *f_x01_* of the same cell. The same procedure for the characterization of *f_x02_* is used for the measurement of *f_x01_*. We turn on the generator and we apply a sinusoidal signal at 10 kHz in order to place the cell in its nDEP behavior. Next, we increase the frequency by steps of 10 kHz until we observe the cell motion. Then, we scan slowly the frequency by steps of 1 kHz to have an accurate value of the crossover frequency. This characterization process is duplicated to confirm the measured value. Finally, the electric signal is turned off and the flow pressure at the chip inlet is increased to release the characterized cell and renew the cell suspension in the microfluidic channel. A new cell is next trapped and fully characterized following the same method.

Hence, the resolution of the two measured crossover frequencies *f_x01_* and *f_x02_* is, respectively, 1 kHz and 1 MHz. One should notice that due to the natural biological heterogeneity occurring among a cell population, the crossover frequencies might spread out on a more or less large frequency range. Nevertheless, the repeatability and reproducibility of the crossover frequency measurements allow us to consolidate the collected data. Afterwards, the comparison of different crossover frequencies recorded from distinct cells or conditions is validated with statistical analyses. Hence, we consider that the identification of the DEP signature (collection of crossover frequencies from distinct tumor cells) is representative of the whole cell population.

### 2.6. Statistical Analysis

Statistical analysis was performed using PAST software. Comparisons between groups were analyzed by ANOVA test. *p* < 0.005 was considered significant (* *p* < 0.05; ** *p* < 0.01; *** *p* < 0.001)

## 3. Results

### 3.1. Enrichment of CSC in the Define Medium

**Figure 7:**
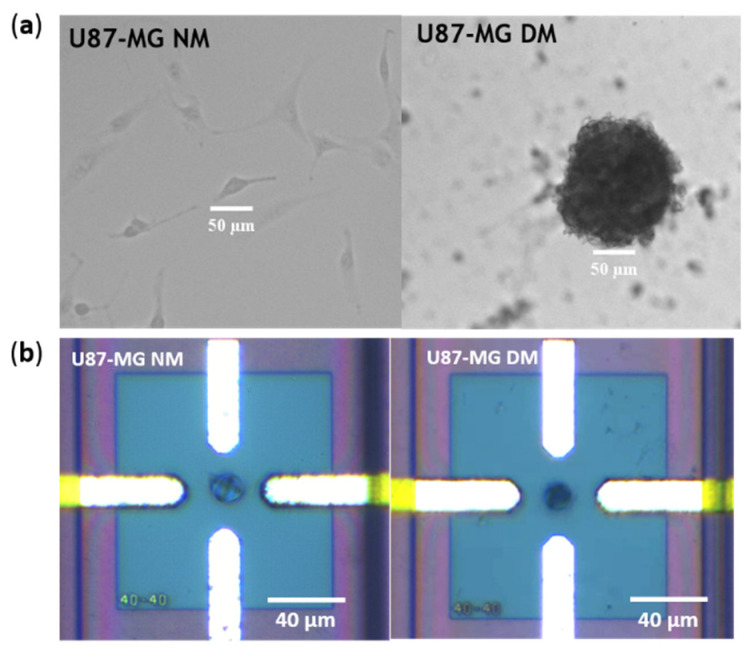
(**a**) Microscope view of the U87-MG cell line cultured in two different conditions: Normal Medium (NM) and after 2 days in define medium (DM); (**b**) microscope view of characterized U87-MG cell line trapped in our quadrupole sensor.

In order to enrich the tumor cell populations in undifferentiated cells related to CSC, U87-MG cells were cultured in Define Medium for 5 days. Morphological changes are observed macroscopically in these stringent culture conditions. As expected, the morphology of U87-MG NM vs. U87-MG DM is completely different (Figure 7a). In Normal Medium, cells are spread out in the petri dish, whereas in Define Medium, cells develop the ability to form glioma spheres due to the presence of specific growth factors (EGF and bFGF-2). It is known that neural stem cells cultured in vitro have the capability to generate clonal structures called “neurospheres” [26]. Glioma spheres are composed of a wealth of aggregated cells. However, just before the DEP characterization, cells are resuspended in the DEP medium and glioma spheres are mechanically broken with the action of a micropipette. When the cell suspension is injected in the lab-on-a-chip, the cells cultured in different conditions present a round shape, and no significant difference in morphology can be observed under an optical microscope (Figure 7b).

**Figure 8:**
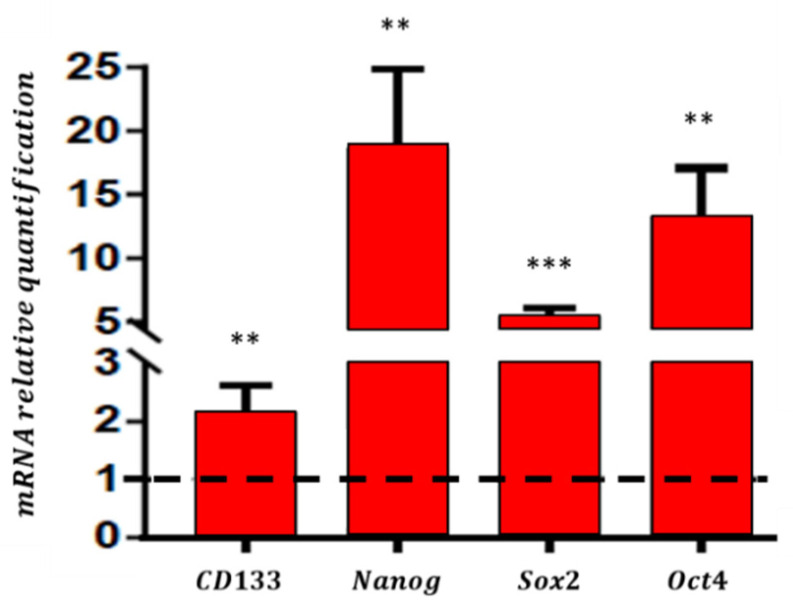
Comparative analysis of gene expression of four undifferentiated markers: *CD*133, *Nanog*, *Sox*2 and *Oct*4 among U87-MG cell line, cultured in Normal Medium (dotted line), or in define medium, measured by Real Time PCR. *** represents *p*-value < 0.001, ** represents *p*-value < 0.01.

To confirm the enrichment of cancer stem cells from total cell population, we achieved a transcriptomic analysis in order to assess the changes of mRNA expression levels related to CSC biomarkers (*CD133*, *Nanog*, *Sox2* and *Oct4*) when U87-MG cells were cultured either in the Normal Medium or in define medium. mRNA relative quantification of U87-MG cultured in Define Medium were normalized, respectively, to the gene expression of U87-MG culture in the Normal Medium (dotted line) (Figure 8). As expected, CSC transcripts were overexpressed in define medium cultured cells, confirming the enrichment of CSCs in cell subpopulation.

### 3.2. Dielectrophoretic Signatures f_x01_ and f_x02_ of U87-MG Cell Line

The U87-MG cell line has been characterized using the microfluidic lab-on-a-chip using the method previously described. The first crossover frequency *f_x01_* and the second crossover frequency *f_x02_* of the same trapped cells have been successively measured.

**Figure 9:**
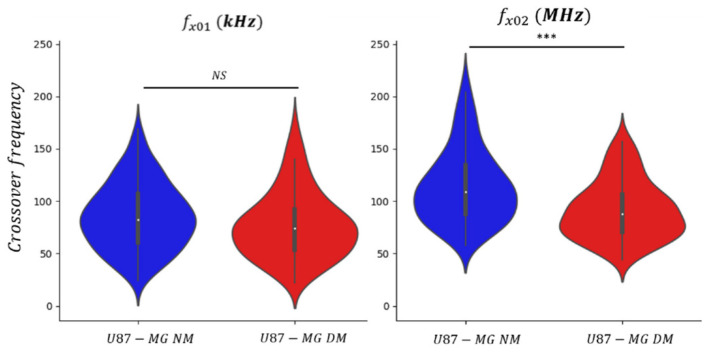
Graphic violin plot representation of U87-MG cells crossover frequencies *f_x01_* (left graph) and *f_x02_* (right graph), cultured in two different conditions: Normal Medium (NM) and define medium (DM). *** represents *p*-value < 0.0001.

The measurement results for both culture conditions summarized in the violin plot thereby illustrate the distribution over frequency of the data (Figure 9). Violin plots are very similar to box plots, except that they additionally show the probability density curve of the different data. The small white dot marker labels the median value of the dataset (small white dot). Moreover, as for the box plot, the first and fourth quartiles of the dataset are represented by the thin black line, and 50% of the whole cell population is concentrated in the thick black line. The extreme peaks correspond to the minimum and maximum values. In Figure 9, one should notice that the scales for the two crossover frequencies are the same except that the unit are kHz and MHz for *f_x01_* and *f_x02_*, respectively.

Descriptive statistics including number of cells, median value and standard deviation of crossover frequency for distinct culture conditions are reported in Table 2. One can notice, for both crossover frequencies datasets on Figure 9, the wide-ranging dispersion of measured values, which is also reflected in the value of the standard deviation. This observation is due to the normal heterogeneity of the cells among the GBM cell population. At low frequency, *f_x01_* is influenced by the small difference of the cell size and morphology within the cell culture, while at high frequency, *f_x02_* might be dependent of intracellular changes or alterations. For instance, during the cell cycle, the nucleocytoplasmic ratio might differ from a cell to another as they are not synchronized during the culture [27]. However, the violin plot highlights the fact that cell’s crossover frequencies are gathered around their respective median value.

A representative number of cells have been individually characterized to statistically consolidate the collected dataset and make the established signatures significant. One can notice that the distribution of the first crossover frequency *f_x01_* is mostly the same for the two culture conditions, NM vs. DM. The median value of *f_x01_* for the undifferentiated enriched population (DM) shows no significant difference with the normal conditions: 74 and 82 kHz, respectively. Indeed, as shown previously in Figure 8, both U87-MG NM and U87-MG DM present the same round shape morphology. In contrast, the distribution of the second crossover frequency *f_x02_* exhibits a significant difference despite an overlap in frequency. This can be explained by the fact that GBM cell population cultured in normal conditions include a majority of differentiated cells but also few undifferentiated cells. However, for the DM conditions, there are more undifferentiated cells, since presenting stringent survival conditions, DM is more selective. Thus, we can observe a decrease in the crossover frequency *f_x02_* with the presence of the DM cell pool. The median values for U87-MG NM and U87-MG DM are, respectively, 109 and 88 MHz. The decrease in *f_x02_* shows a significant difference between the two cells’ phenotypes, as the p-value is lower than 10^−3^.

This demonstrates that undifferentiated cells compared to differentiated cells own different intracellular dielectric properties. Despite displaying two crossover frequencies, only the one in the UHF range, *f_x02_*, is sufficiently meaningful to be exploited for identifying cells presenting an undifferentiated state or a stemness-like phenotype. These results show how promising UHF-DEP cell profile analysis might be for the discrimination of cell subpopulation within the tumor. Next, we will focus on the second crossover frequency *f_x02_* and strengthen the relevance of using UHF-DEP as a discriminant parameter through a kinetic study of the evolution of the stemness phenotype.

### 3.3. Kinetic Evolution of the Stemness Phenotype

As the enrichment of this cell population is accomplished by seeding normal U87-MG cells in Define Medium, either cells already exerting an undifferentiated profile can survive, or other cells must acquire phenomenon is a process that requires several division cycles and we specific features related to stem cells to survive in Define Medium. With the acquirement of cell undifferentiated status, cell aggressiveness is increased and is related to tumor aggressiveness. The undifferentiation proposed to follow its kinetics by UHF-DEP to demonstrate the potential of the UHF-DEP microsystem developed.

**Figure 10:**
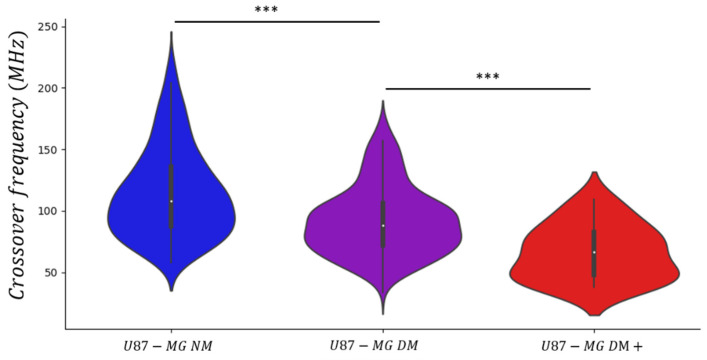
Graphic violin plot representation of U87-MG cells crossover frequency *f_x02_*, cultured in three different conditions: (i) 5 days in Normal Medium (NM); (ii) 5 days in define medium (DM); (iii) 21 days in define medium (DM+). *** represents *p*-value < 0.001.

To do so, the U87-MG cell line was cultured within three different conditions: (i) 6 days in Normal Medium (NM); (ii) 5 days in Define Medium (DM); (iii) maintained during 21 days in Define Medium (DM+). The results of the measured crossover frequency *f_x02_* are presented in the violin plot chart in Figure 10. In addition to the data already collected previously for U87-MG NM and U87-MG DM cells, we remeasured about hundred more crossover frequencies *f_x02_* in order to consolidate the previously obtained DEP signatures and to improve the statistical strength of our analysis.

Statistics results related to the collected data are reported in Table 3, including the median value and the standard deviation of *f_x02_*. As said before, the distribution of the second crossover frequency values is dispersed. This observation is highlighted by the high values of the standard deviation. However, the standard deviation seems to decrease the more the cells are maintained in Define Medium. Indeed, as the GBM cell line presents a high biological heterogeneity, the Define Medium tends to select only cells which are able to survive under such stringent culture conditions, i.e., cells with an undifferentiated phenotype. Despite the data dispersion, the violin plot shows that the cell’s crossover frequency values are mostly gathered around their respective median value.

In Normal Medium, the median value of the second crossover frequency is 108 MHz. After 5 days in Define Medium, the median DEP signature decreases to 88 MHz and after 16 additional days in Define Medium, it is 67 MHz. The two successive decreases in *f_x02_* observed between the three cell populations present significant differences, as the *p*-value is lower than 10^−3^. In correlation with the previous experimentation, a lower dielectric signature seems to potentially characterize cells presenting a stemness-like phenotype.

One can also notice that although more cells were characterized in the NM and DM conditions for this measurement campaign, the median value of the crossover frequency is not affected and remains the same compared to the first campaign (related in Table 2). It shows the robustness and the reproducibility of our method to measure the DEP signatures of cells.

These two experiments demonstrate the ability of the developed UHF-DEP lab-on-chip to successfully extract information about the potential stemness status of U87-MG cells by the measurement of the second crossover frequency *f_x02_*.

### 3.4. Dielectrophoretic Signatures f_x01_ and f_x02_ of GBM Primary Cultures

We previously measured (in Section 3.2) the low- and high-frequency DEP signatures of the U87-MG cell line, cultured in two different conditions in order to induce an undifferentiation corresponding to the CSC subpopulation. From the obtained crossover frequency, *f_x01_* did not show any difference between Normal Medium and Define Medium. However, we demonstrated that *f_x02_* presents a significant difference and can be a relevant discriminant parameter to identify the CSC subpopulation. These results of the DEP signatures were obtained from the in vitro cell line.

To go further, we proposed in the last section of this paper to repeat this experiment on ex vivo GBM primary cultures to demonstrate the potential clinical applications of our approach.

The two crossover frequencies *f_x01_* and *f_x02_*, respectively were characterized in GBM primary culture cells derived from four patients. These cells were collected after surgery on patients suffering from glioblastoma. Once extracted from the tumor samples, cells were put in culture according to the procedure indicated in Materials and Methods. As for the U87-MG cell line, a few minutes before DEP characterization, the ex vivo GBM cells were resuspended in the DEP medium. Then, for each investigated cell, their two crossover frequencies *f_x01_* and *f_x02_* were measured with the previously described protocol.

**Figure 11:**
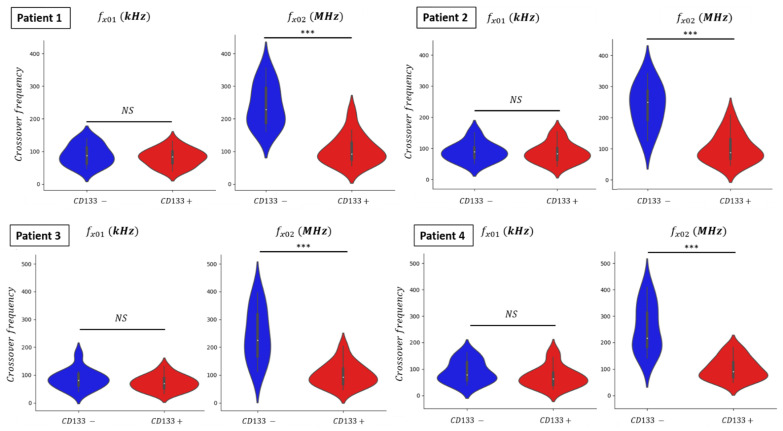
Graphic violin plot representation of crossover frequencies *f_x01_* and *f_x02_* of GBM primary cells collected from four different patients. *** represents *p*-value < 0.001.

Before DEP characterizations, the GBM cell population was first separated into two subpopulations: CD133− and CD133+. CD133 is a transmembrane protein expressed in human hematopoietic stem cells and progenitor cells [28]. As said before, CD133 is a biomarker associated with stem-like cells, and thus with tumor regeneration. It is possible to mark cells with monoclonal antibodies anti-AC133 coupled with a fluorochrome to detect the presence of the peptide CD133 on the cell surface [29]. Nevertheless, CD133 can be also expressed in differentiated cancer cells, so the whole cell population will present a fluorescent intensity gradient [30]. Hence, we impose a threshold of the fluorescent intensity during the passage of cells in the flow cytometer to define two subpopulations. The CD133+ population is the cell population that overexpresses the marker and thus is enriched in CSCs, while the CD133− population is the population of differentiated cells [28]. Therefore, we separate and isolate the CSC cell population from the differentiated one thanks to a fluorescent marker, before DEP characterizations of these populations. The results of the measured crossover frequencies *f_x01_* and *f_x02_* for both populations are presented in the violin plot (Figure 11).

One should notice that the scale for the two crossover frequencies is the same except that the units are kHz and MHz for *f_x01_* and *f_x02_*_,_ respectively. The data distribution of crossover frequencies *f_x01_* and *f_x02_* from the characterized glioblastoma cell shows similar violin plot shapes although cells were derived from four different patients (Figure 11). Indeed, we can expect from ex vivo GBM cells to be even more genetically heterogeneous from one patient to another than the immortalized U87-MG cell line. Median values of the crossover frequencies measured for each isolated population are reported in the Table 4. The obtained results highlight that our DEP cell analyzer lab-on-chip is a relevant and reliable tool to study and analyze either in vitro or ex vivo dissociated samples.

The distribution of the first crossover frequency *f_x01_* is mostly the same for the two isolated populations, CD133− vs. CD133+. The median value of *f_x01_* for the undifferentiated population (CD133+) shows no significant changes with the differentiated one. At low frequency, the largest signature dissimilarity between the two conditions corresponds to patient 4, where *f_x01_* displays a change of 15 kHz between CD133− and CD133+ conditions (from 78 to 63 kHz). However, this difference is not statistically significant to discriminate the subpopulation of CSCs overrepresented in the CD133+ cells. The distribution of the second crossover frequency *f_x02_* exhibits a difference despite an overlap among the measured values. This can be explained by the method of enrichment through flow cytometry. Indeed, the CD133 biomarker is not a binary label as the whole cell population might present different fluorescent intensities. With this fluorescent gradient, we choose a threshold to separate GBM cells into two subpopulations and to be selective toward CSC population. Thus, we can observe a decrease in the crossover frequency *f_x02_* with the presence of the CD133+ cell pool. The median values of the second crossover frequency display the smallest change for patient 4, which is 125 MHz (from 216 to 91 MHz). The decrease in *f_x02_* shows a significant difference between the two cell populations, as the p-value is lower than 10^−3^. Moreover, one can notice in Figure 11 that at high frequency, the CD133+ cell population displays a stoutness around the median value compared to the CD133− cell, for whom the values are gathered around their median.

Such results validate that we can exploit the intracellular dielectric properties differences between differentiated and undifferentiated cells by measuring the second crossover frequency *f_x02_*. The cells extracted from patients’ GBM tumor samples show similar behavior as observed with an in vitro GBM cell line. The *f_x02_* median value of ex vivo cells are not the same as in vitro results, but we can extrapolate that an ex vivo cell with a low *f_x02_* could be a stem-like cell.

## 4. Conclusions

In this article, we used an innovative microfluidic device based on high-frequency dielectrophoresis for single-cell characterization in order to discriminate and identify the cancer stem cell subpopulation. First, we evaluated the discrimination capabilities of our microfluidic device in vitro on a glioblastoma cell line. U87-MG cells were cultured in two distinct conditions: one inducing differentiation and the second selecting immature and undifferentiated cells. Our results suggest that the expression of biological CSC markers and the measurement of the UHF crossover frequency *f_x02_* are closely linked. At this frequency range, our lab-on-chip is able to interact with the intracellular content, which is more representative of the undifferentiated features of cells, making UHF-DEP greatly relevant to investigate the stemness status of cancer cells. As a first step towards clinical experiments, some GBM cells were extracted and cultured from patients’ tumors. These GBM primary cells have been sorted into two subpopulations according to their expression level of CSC biomarker CD133. Whatever the considered patient, observed DEP signatures display the same profile. As previously identified, *f_x02_* shows a more significant and more important difference between the two cell phenotypes than *f_x01_* and so is confirmed to be a relevant CSC discriminant parameter. As primary culture cells are more representative of tumor than cell lines, we believe that it might be possible to transpose this capability of UHF-DEP cell characterization for recognizing “stemness” features from tumor cells derived to a broad range of GBM patients.

UHF-DEP is a very promising tool with great potential to discriminate cells according to their internal biological properties. Hence, from the identification of UHF-DEP signatures, we can see the perspective to develop a cell sorting device for isolating cancer stem cells [31]. In the future, the early detection of CSC subpopulation in a glioma tumor with a UHF-DEP approach could have a prognosis value on therapeutic response and might allow adaption of a therapeutic strategy following diagnosis.

## Figures and Tables

**Table 1:** Values of the different dielectric and cellular parameters used in the COMSOL Multiphysics simulation.

Parameter	Value
Particle radius	11.5 µm
Membrane thickness	700 nm ^1^
Intracellular relative permittivity	50
Intracellular conductivity	0.5 S/m
Membrane relative permittivity	100^2^
Membrane conductivity	1.43 × 10^4^ S/m ^2^
Medium relative permittivity	78
Medium conductivity	0.02 S/m

^1^ Membrane thickness was increased by 100 in order to avoid mesh issues during the computation. ^2^ Data were modified proportionally due the modification of the membrane thickness in order to respect the cell dielectric behavior.

**Table 2:** Values regarding the crossover frequency measurements of the U87-MG cell line.

Cell Culture Conditions	Crossover Frequency	Number of Cells Measured	Median Value	SD
Normal Medium (NN)	*f_x01_*	139	82 kHz	31.5 kHz
Define medium (DN)		134	74 kHz	32.1 kHz
Normal Medium (NN)	*f_x02_*	139	109 MHz	35.2 MHz
Define medium (DN)		134	88 MHz	27.9 MHz

**Table 3:** Values regarding the crossover frequency measurements of the U87-MG cell line.

Culture Condition	Median Value	SD
Normal Medium (NM)	108 MHz	36.2 MHz
Define Medium (DM)	88 MHz	27.9 MHz
Define Medium (DM+)	67 MHz	22.1 MHz

**Table 4:** Values of the crossover frequency measurements of the GBM primary culture extracted from tumor samples of four different patients.

	Cell Population	Crossover Frequency	Median Value
**Patient 1**	CD133−	*f_x01_*	88 kHz
	CD133+		83 kHz
	CD133−	*f_x02_*	229 MHz
	CD133+		92 MHz
**Patient 2**	CD133−	*f_x01_*	89 kHz
	CD133+		83 kHz
	CD133−	*f_x02_*	248 MHz
	CD133+		86 MHz
**Patient 3**	CD133−	*f_x01_*	81 kHz
	CD133+		70 kHz
	CD133−	*f_x02_*	225 MHz
	CD133+		92 MHz
**Patient 4**	CD133−	*f_x01_*	78 kHz
	CD133+		63 kHz
	CD133−	*f_x02_*	216 MHz
	CD133+		91 MHz
